# Impaired Regulated Exocytosis and GLUT4 Trafficking in Dysferlin-Deficient Human Myoblasts: A Pilot Study

**DOI:** 10.3390/biomedicines14102207

**Published:** 2026-09-29

**Authors:** María José Guerra-Fernández, Walter Vásquez, Hugo Almarza-Salazar, Constanza Bertea-Seissus, Sissi Fuentealba, María Constanza Maldifassi, Pablo Caviedes, Arlek Gonzalez-Jamett, Ana M. Cardenas

**Affiliations:** 1 Instituto Milenio Centro Interdisciplinario de Neurociencia de Valparaíso, Valparaíso2360102, Chile; mjguerraf@gmail.com (M.J.G.-F.); wavasqueza@gmail.com (W.V.); haalmarza@uc.cl (H.A.-S.); constanza.bertea@gmail.com (C.B.-S.); sissi.fuentealba@gmail.com (S.F.); maria.maldifassi@uv.cl (M.C.M.); arlek.gonzalez@uv.cl (A.G.-J.); 2 Instituto de Fisiología, Facultad de Ciencias, Universidad de Valparaíso, Valparaíso 2360102, Chile; 3 Núcleo Interdisciplinario de Farmacología e Inmunología, Instituto de Ciencias Biomédicas, Facultad de Medicina, Universidad de Chile, Santiago 2360102, Chile; pcaviedes@ing.uchile.cl; 4 Centro de Biotecnología y Bioingeniería (CeBiB), Departamento de Ingeniería Química, Biotecnología y Materiales, Facultad de Ciencias Físicas y Matemáticas, Universidad de Chile, Santiago 8389100, Chile; 5 Centro para la Investigación Traslacional en Neurofarmacología, CItNe, Universidad de Valparaíso, Valparaíso 2360102, Chile; 6 Escuela de Química y Farmacia, Facultad de Farmacia, Universidad de Valparaíso, Valparaíso 2360102, Chile; 7 Instituto de Neurociencia, Facultad de Ciencias, Universidad de Valparaíso, Valparaíso 2360102, Chile

**Keywords:** exocytosis, dysferlin, skeletal muscle, dystrophy, myoblasts, GLUT4, dysferlinopathy

## Abstract

**Background/Objectives**: Dysferlinopathies are inherited muscular dystrophies caused by DYSF mutations that lead to progressive muscle degeneration. Proteomic analyses of patient muscle reveal altered abundance of proteins involved in regulated exocytosis and energy metabolism, reflecting complex molecular remodeling within these pathways. However, whether dysferlin deficiency impairs GLUT4 exocytosis remains unknown. **Methods**: Immortalized human myoblasts from a healthy control (C25) and a dysferlinopathy patient (DYSF2) were used. Cytosolic Ca^2+^ was measured using Fura-2 and Fluo-4; submembrane Ca^2+^ dynamics were recorded with membrane-targeted sensor pN1-Lck-GCaMP3. Single exocytosis events induced by 10 µM ionomycin or 100 nM insulin were tracked with IRAP-pHluorin and total internal reflection fluorescence microscopy (TIRFM). GLUT4 puncta near the plasma membrane were quantified using immunofluorescence and visualized by TIRFM. Dysferlin-3HA was expressed to test rescue. **Results**: Both cell lines showed comparable cytosolic and submembrane Ca^2+^ responses to ionomycin. However, the frequency of IRAP-pHluorin exocytosis events evoked by both ionomycin and insulin was significantly reduced in DYSF2 myoblasts. Expression of dysferlin-3HA restored insulin-induced exocytosis in DYSF2 cells to control levels but failed to fully rescue ionomycin-induced events. Insulin increased the trafficking of GLUT4 puncta near the plasma membrane in C25 cells, whereas DYSF2 cells lacked this insulin-stimulated response. **Conclusions**: In the patient-derived cell line examined here, dysferlin deficiency impaired stimulus-evoked exocytosis and insulin-induced GLUT4 translocation without altering Ca^2+^ dynamics. The inability of dysferlin-3HA to significantly rescue ionomycin-induced exocytosis points to additional downstream defects, likely secondary proteomic alterations, that constrain full functional recovery.

## 1. Introduction

Dysferlinopathies are progressive muscular dystrophies caused by mutations in the DYSF gene and are characterized by the absence or reduced expression of dysferlin protein and progressive muscle atrophy [1]. Dysferlin is a transmembrane protein with seven cytosolic C2 domains that is essential for sarcolemmal repair [1] and also participates in other Ca^2+^-dependent fusion processes, including lysosomal exocytosis in myoblasts and endothelial cells [2,3]. In vitro studies suggest that dysferlin promotes membrane fusion by facilitating Ca^2+^-dependent assembly of the soluble N-ethylmaleimide-sensitive factor attachment protein receptor (SNARE) complex [4]. Additionally, dysferlin interacts with annexin A2 [5], a Ca^2+^-binding protein that organizes lipid microdomains for vesicle docking and fusion [6].

Proteomics of muscle from patients diagnosed with dysferlinopathy reveals increased abundance of proteins involved in regulated exocytosis, alongside reduced expression of proteins related to energy metabolism and Ca^2+^ transmembrane transport [7]. These data suggest that defects in exocytosis, Ca^2+^ homeostasis and energy metabolism drive compensatory mechanisms aimed at preserving sarcolemmal integrity and cellular metabolism. Consistent with these observations, murine dysferlinopathy models exhibit marked metabolic dysregulation in quadriceps, including altered glucose handling, glycogen accumulation, and mitochondrial abnormalities [8].

Regulated exocytosis in skeletal muscle is critical for glucose homeostasis and depends on insulin- and Ca^2+^-stimulated translocation of GLUT4-containing vesicles (GSVs) and their SNARE-mediated fusion with the plasma membrane [9]. How dysferlinopathy affects insulin- or Ca^2+^-triggered exocytosis of GSVs remains unknown. To address this, we tracked single exocytotic events in immortalized myoblasts from a dysferlinopathy patient (DYSF2 cells) and a healthy control (C25 cells) using insulin-regulated aminopeptidase (IRAP) fused to the pH-sensitive reporter pHluorin. IRAP is a transmembrane protein expressed in GSVs [10,11] that participates in their trafficking [12,13]. By combining the IRAP-pHluorin reporter with total internal reflection fluorescence microscopy (TIRFM), we achieved a direct, real-time readout of GSV fusion events. Integrating this approach with live-cell imaging of cytosolic and submembrane Ca^2+^ signals enabled a comprehensive characterization of how GLUT4 trafficking is affected in human dysferlinopathy.

## 2. Materials and Methods

### 2.1. Culture of Cell Lines

C25 and DYSF2 cells were obtained from the platform for the immortalization of human cells at the Institut de Myologie (Paris, France), with agreement from the subjects through the signing of an informed consent form and anonymization before immortalization, according to the EU’s General Data Protection Regulation. The C25 cell line was established from a biopsy of the semitendinosus muscle of an unaffected individual [14]. DYSF2 was established from human biopsies of patients bearing compound heterozygous c.855+1delG/c.895G>A mutations [15]. These cells were cultivated at a density of 3 × 10^5^ cells/mL in 25 mm glass coverslips in 4:1 Dulbecco’s modified Eagle minimal essential medium/199 medium (DMEM/M199; Sigma-Aldrich St. Louis, MO, USA) supplemented with 20% fetal bovine serum, 25 μg/mL fetuin (Sigma-Aldrich, St. Louis, MO, USA), 0.5 ng/mL basic fibroblast growth factor (Gibco BRL, Gaithersburg, MD, USA), 5 ng/mL epidermal growth factor (ibco BRL, Gaithersburg, MD, USA), 0.2 μg/mL dexamethasone (Sigma-Aldrich, St. Louis, MO, USA), 5 μg/mL insulin (Eli Lilly Co., Indianapolis, IN, USA), 50 U/mL penicillin (OPKO, Santiago, Chile), and 100 µg/mL gentamicin (Gibco/Life Technology, Shanghai, China), and incubated at 37 °C in a 5% CO_2_ atmosphere.

### 2.2. Cytosolic Ca^2+^ Measurements

Cells were loaded with 5 μM Fluo-4 AM or 5 μM Fura-2 AM (Invitrogen, Eugene, OR, USA) in serum-free DMEM for 30 min at 37 °C. After loading, cells were washed three times and maintained in HEPES-buffered extracellular solution (HBECS) containing (in mM): 140 NaCl, 5.9 KCl, 1.2 MgCl_2_, 2 CaCl_2_, 10 D-glucose, 10 HEPES-NaOH, pH 7.4, at room temperature (20 ± 2 °C) for the duration of the recordings. Ca^2+^ transients were evoked with ionomycin (Invitrogen, Carlsbad, CA, USA) at 10, 20, or 40 μM, prepared in the same extracellular solution.

Fluo-4 fluorescence was imaged with a 40× objective on a Nikon Eclipse Ti-E microscope (Nikon, Tokyo, Japan) using a 488 nm laser and an ORCA-Flash 2.0 camera (Hamamatsu, Shizuoka, Japan). Images were acquired at 3.3 Hz for 3 min with NIS-Elements AR 4.3 (Nikon, Tokyo, Japan). Fluo-4 signals are reported as F/F_0_ (fluorescence normalized to baseline) after background subtraction.

Fura-2 recordings were obtained on a Nikon Eclipse Ti microscope (Nikon, Tokyo, Japan) with alternating excitation at 340 and 380 nm and emission collected at 510 nm. Images were captured every 3 s using NIS sofware 4.3 (Melville, NY, USA). Ca^2+^ signals are presented as the 340/380 fluorescence ratio and quantified as ΔR/R_0_.

### 2.3. Measurement of Submembrane Ca^2+^ Signals

To monitor submembrane Ca^2+^, cells were transfected with pN1-Lck-GCaMP3 (Addgene plasmid #26974) [16] using Lipofectamine 2000 (Invitrogen, Carlsbad, CA, USA). Imaging was performed 24 h after transfection. During imaging, cells were bathed in HBECS and maintained at room temperature (20 ± 2 °C). Fluorescence responses to 10 μM ionomycin were recorded at 3.3 Hz for 3 min on a Nikon Eclipse Ti-E microscope (Tokyo, Japan) with a 100× APO TIRFM objective (NA 1.49) and 488 nm laser and were acquired with an ORCA-Flash 2.0 camera (Hamamatsu, Shizuoka, Japan); pixel size 6.5 × 6.5 µm). Data are expressed as F/F_0_ (fluorescence normalized to resting conditions) with background subtraction.

### 2.4. Monitoring of Single Exocytosis Events

To detect single exocytic events, cells were transfected with IRAP-pHluorin (kindly gifted by Dr. J. Lippincott-Schwartz) [17] using Lipofectamine 2000 (Invitrogen, Carlsbad, CA, USA). Imaging was conducted 48 h post-transfection in the HBECS at room temperature (20 ± 2 °C). Exocytosis events induced with 10 μM ionomycin or 100 nM insulin were recorded and acquired using the imaging configuration described in Section 2.3. Events were analyzed using custom Python-3-based software on the Anaconda platform. This software automatically identified exocytosis as fluorescent spots (2–50 pixels in diameter) with a ΔF of at least 3 standard deviations above baseline [18]. Durations were computed in Python 3 using NumPy, SciPy and standard math/statistics libraries. For each event we identified the pre-rise baseline frame, the first frame showing a sustained fluorescence increase, and the frame at which the trace returned to baseline [18].

### 2.5. Dysferlin-3HA Expression and Immunofluorescence

For rescue experiments, DYSF2 myoblasts were transfected with 1 µg of a dysferlin-3HA plasmid construct (Addgene plasmid #29767; provided by The Jain Foundation) using Lipofectamine 2000 (Invitrogen, Carlsbad, CA, USA) according to the manufacturer’s instructions. Expression of the construct was confirmed by immunofluorescence; briefly, cells were fixed with 4% paraformaldehyde for 10 min at room temperature, washed with PBS, permeabilized with 0.1% Triton X-100 for 10 min, and blocked with 1% bovine serum albumin in PBS. Cells were then incubated overnight at 4 °C with a rabbit anti-dysferlin primary antibody (Abcam, California, USA; ab124684; 1:300 dilution), washed three times with PBS, and incubated for 1 h at room temperature with a Cy2-conjugated anti-rabbit secondary antibody (Jackson ImmunoResearch, West Grove, PA, USA; 1:1000 dilution). Following final washes in PBS, fluorescence images were acquired using a Leica Stellaris 5 LIA + RS spectral confocal microscope equipped with a 40× objective (Leica Microsystems, Mannheim, Germany).

### 2.6. Measurement of GLUT4 Surface-Proximal Expression

C25 and DYSF2 myoblasts were seeded on 25 mm coverslips and maintained in DMEM/M199 supplemented with 20% fetal bovine serum, 50 U/mL penicillin and 100 µg/mL gentamicin. After three days, cells were stimulated at 37 °C with 100 nM insulin for 30 min in HEPES-buffered Krebs solution, containing in mM: 118 NaCl, 4.7 KCl, 2 CaCl_2_, 1.2 MgCl_2_, 10 D-glucose, 10 HEPES pH 7.4. After stimulation, cells were fixed with 4% paraformaldehyde for 10 min at room temperature, washed with phosphate-buffered saline (PBS), permeabilized with 0.1% Triton X-100 for 10 min, and blocked with 1% bovine serum albumin in PBS. Samples were incubated overnight at 4 °C with anti-GLUT4 primary antibody (Santa Cruz Biotechnology, CA, USA; 1:100). After three PBS washes, cells were incubated with Cy2-conjugated anti-mouse secondary antibody (Jackson ImmunoResearch; 1:500) for 1 h at room temperature. Following additional PBS washes, images were acquired by TIRFM with a 100× oil objective using the acquisition settings described above.

Particle density proximal at the plasma membrane was quantified in Fiji (ImageJ 1.54p) after background subtraction and thresholding using the Particle Analysis tool. Total particle counts were normalized to cellular area, determined by manually outlining cell boundaries in brightfield images; nuclear regions were excluded from analysis to avoid non-specific nuclear fluorescence.

### 2.7. Statistical Analysis

For each condition, data were obtained from three different cell cultures, with a single measurement performed per cell. No test for outliers was performed. Normality was assessed using the Shapiro–Wilk and D’Agostino–Pearson tests. For normally distributed data, comparisons between two independent groups were performed using a two-tailed Student’s *t*-test; comparisons among multiple independent groups used a one-way ANOVA followed by Tukey’s post hoc test. Non-normally distributed data involving multiple groups were analyzed using the Kruskal–Wallis test followed by Dunn’s post hoc test. Statistical analyses were conducted using Prism software version 5 (GraphPad V5 Software Inc., La Jolla, CA, USA). Data are expressed as means ± SEM, and a significance level of *p* < 0.05 was applied.

### 2.8. Ethics Statement

This research was approved by the Biosafety and Bioethics committees of Universidad de Valparaíso (Chile), with approval identification numbers BS015-22 and BEA188-22, respectively.

## 3. Results

### 3.1. Cytosolic and Submembrane Ca^2+^ Signals Are Not Affected in DYSF2 Myoblasts

In human myoblasts, Ca^2+^ signals regulate diverse physiological processes, including exocytosis, cytokine production, and cellular differentiation [18,19]. To investigate these signals in the context of dysferlinopathy, we compared cytosolic Ca^2+^ dynamics between C25 cells (derived from an unaffected individual) [14] and DYSF2 cells (derived from a patient with dysferlinopathy) [15], which exhibit low dysferlin expression [15,20].

To trigger Ca^2+^ signals, we utilized 10 µM ionomycin, a Ca^2+^ ionophore previously shown to effectively induce cytosolic Ca^2+^ rises in C25 cells [18]. As illustrated in Figure 1A, ionomycin stimulation elicited a rapid and sustained increase in cytosolic Ca^2+^ in both C25 and DYSF2 cells. Quantitative analysis revealed no significant differences in the peak response between the two cell lines, reaching similar maximal fluorescence levels (Figure 1A; F/F_0_ = 2.7 ± 0.14 for C25 and 2.9 ± 0.13 for DYSF2). To confirm that Fluo-4 signals were not saturated during ionomycin exposure, we also measured cytosolic Ca^2+^ with the ratiometric dye Fura-2 and applied three ionomycin concentrations (10, 20, and 40 µM). As shown in Figure 1B, these concentrations produced dose-dependent increases in Ca^2+^, with no significant differences between C25 and DYSF2 cells at any concentration.

Furthermore, submembrane Ca^2+^ signals were assessed in C25 and DYSF2 cells expressing pN1-Lck-GCaMP3, a modified Ca^2+^ sensor containing a Lck membrane-tethering sequence [21]. Figure 1C displays TIRFM images of both cell lines before and after ionomycin stimulation. Representative traces of these submembrane Ca^2+^ transients are shown in Figure 1D. Consistent with the cytosolic data, submembrane Ca^2+^ signals reached similar maximal amplitudes in both control and dysferlinopathy myoblasts (Figure 1E; peak F/F_0_ = 2.2 ± 0.2 and 2.6 ± 0.3, respectively).

### 3.2. Ca^2+^-Induced Exocytosis Is Reduced in Myoblasts Derived from a Dysferlinopathy Patient

Exocytosis was assessed using TIRFM in C25 and DYSF2 cells expressing IRAP-pHluorin. The fluorescence of this reporter is quenched in the acidic environment (pH~5.5) within the vesicle lumen but increases abruptly upon fusion with the plasma membrane as it is exposed to the neutral extracellular medium. Thus, these fusion events therefore appear as rapid increases in fluorescence intensity of variable duration [18]. Using our custom Python-3-based software, we detected and analyzed the appearance of new fusion events.

Figure 2A,B display TIRFM images of C25 and DYSF2 cells expressing IRAP-pHluorin under baseline and ionomycin-stimulated conditions. In our prior work, over 90% of fluorescent events in C25 cells were quenched when cells were exposed to pH 5.5 [18].

With pHluorin-tagged membrane proteins imaged by TIRFM, two fluorescence patterns are observed: events that spread laterally as proteins diffuse in the plasma membrane, and events that rapidly dim without lateral spread, consistent with retrieval at the fusion site [22,23,24]. In our prior analysis, ~80% of single exocytotic events exhibited the non-lateral (restricted) pattern [18], as exemplified in Figure 2C; the corresponding fluorescence trace is shown in Figure 2D. During a 3 min recording, the number of new fluorescence spots in cells treated with the vehicle DMSO was 0.023 ± 0.005 and 0.013 ± 0.001 events/µm^2^ in C25 and DYSF2 cells, respectively. Stimulation with 10 µM ionomycin increased the event frequency in C25 myoblasts to 0.347 ± 0.068 events/µm^2^ (*p* < 0.001 compared with DMSO-treated cells), whereas DYSF2 myoblasts showed no significant increase in exocytosis after ionomycin stimulation (Figure 2E).

To compare single-event kinetics, we measured event duration, defined as the time from the frame immediately before the fluorescence rise to the frame when fluorescence returned to baseline. Because some events did not conclude within the recording window, durations were calculated only for events that reached baseline within the acquisition. This analysis showed that ionomycin increased the mean duration of individual exocytotic events in C25 myoblasts (vehicle: 37.4 ± 4.6 s; 10 µM ionomycin: 80.6 ± 7.1 s). No comparable increase was observed in DYSF2 cells (Figure 2F).

### 3.3. Insulin-Induced Exocytosis Is Reduced in Dysferlinopathy Myoblasts

We next evaluated the effect of a physiological stimulus by treating the cells with insulin. Figure 3A,B display TIRFM images of C25 and DYSF2 cells expressing IRAP-pHluorin under resting and insulin-stimulated conditions. A representative example of an individual exocytotic event in a C25 myoblast is illustrated in Figure 3C, with its corresponding fluorescence intensity heat map shown in Figure 3D. Under resting conditions without stimulation, C25 and DYSF2 myoblasts display an average of 0.02 ± 0.003 and 0.01 ± 0.003 events/µm^2^ during the recording period, respectively. Insulin at 100 nM elicited a two-fold increase in the number of exocytotic events in C25 myoblasts but failed to do so in DYSF2 cells (Figure 3E).

Average durations of single exocytosis events induced by insulin are shown in Figure 3F. In C25 cells, event durations were 31.6 ± 2.8 s under resting conditions and 26.9 ± 1.8 s with insulin stimulation. In resting DYSF2 cells, event durations were 12.4 ± 3.5 s, significantly lower than in non-stimulated C25 cells. Upon insulin stimulation, event durations in DYSF2 cells significantly increased to 36.9 ± 4.9 s compared to resting conditions (*p* < 0.001).

Taken together, these findings indicate that dysferlinopathy myoblasts exhibit significant defects in both the frequency and kinetics of exocytosis.

**Figure 2:**
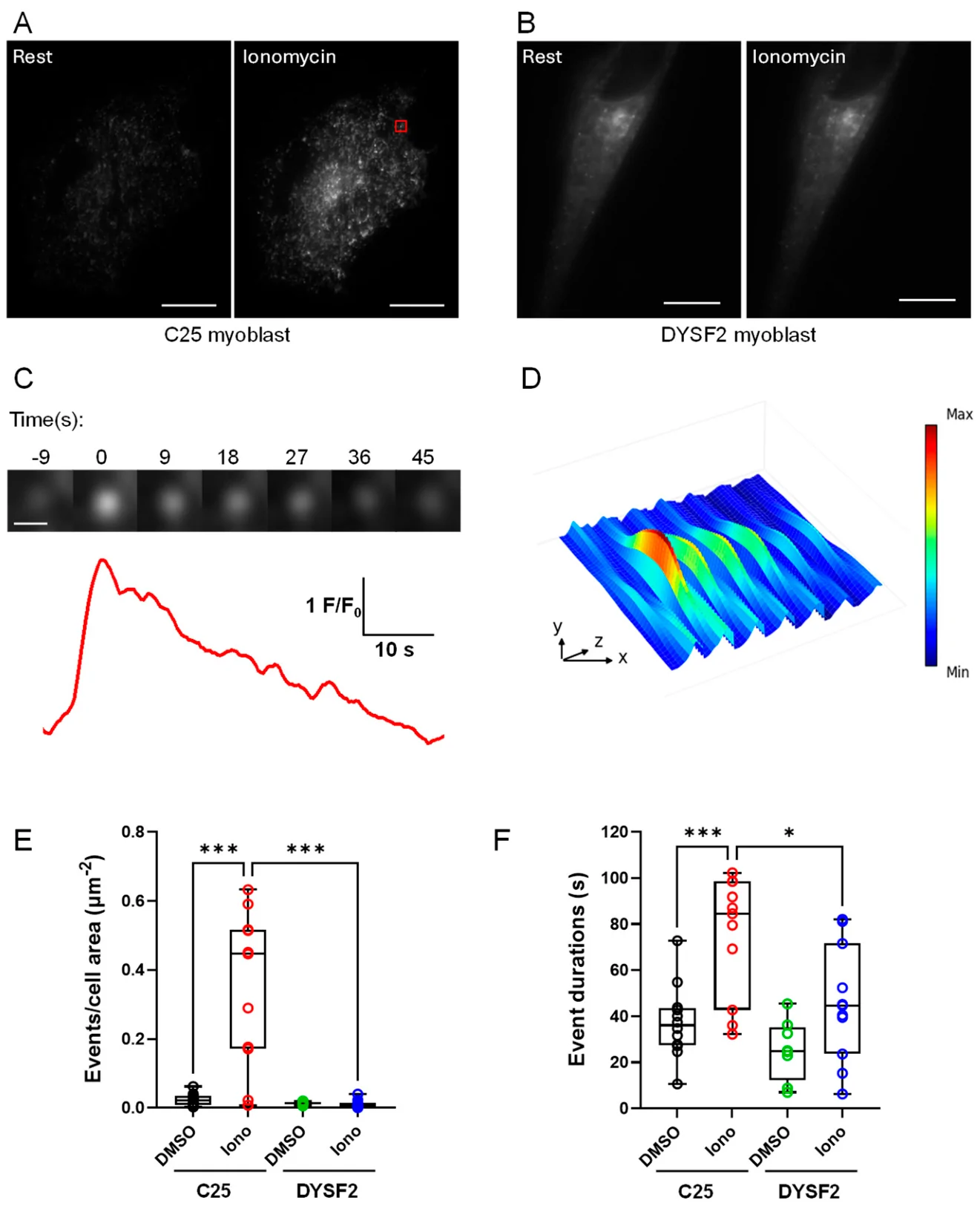
Ionomycin-induced exocytosis is impaired in dysferlinopathy myoblasts. Control C25 and dysferlinopathy DYSF2 myoblasts were transfected with IRAP-pHluorin to monitor exocytosis induced by 10 μM ionomycin. (**A**,**B**) A TIRFM image of a C25 (**A**) and DYSF (**B**) myoblast in resting conditions (**left** panels) stimulated with 10 μM ionomycin (**right** panels). Scale bar = 5 μm. (**C**) **Upper**: Sequence of frames depicting the exocytosis event indicated by the red square in A (**right** panel). Scale bar = 0.15 μm. **Bottom**: Fluorescence intensity profile of the exocytosis event. (**D**) Heat map showing the fluorescence profile of the event displayed in (**C**). The x-, y-, and z-axes represent time, spatial position along the analyzed region, and fluorescence intensity, respectively. (**E**) Comparison of the number of exocytosis events between C25 and DYSF2 myoblasts under vehicle (DMSO) or 10 μM ionomycin stimulation. (**F**) Durations of individual exocytosis events. Data points show values from individual cells across at least three independent cultures, with horizontal lines indicating means and SEM. Statistical significance is indicated as * *p* < 0.05, *** *p* < 0.001 (ANOVA test followed by Tukey’s multiple comparison test).

**Figure 3:**
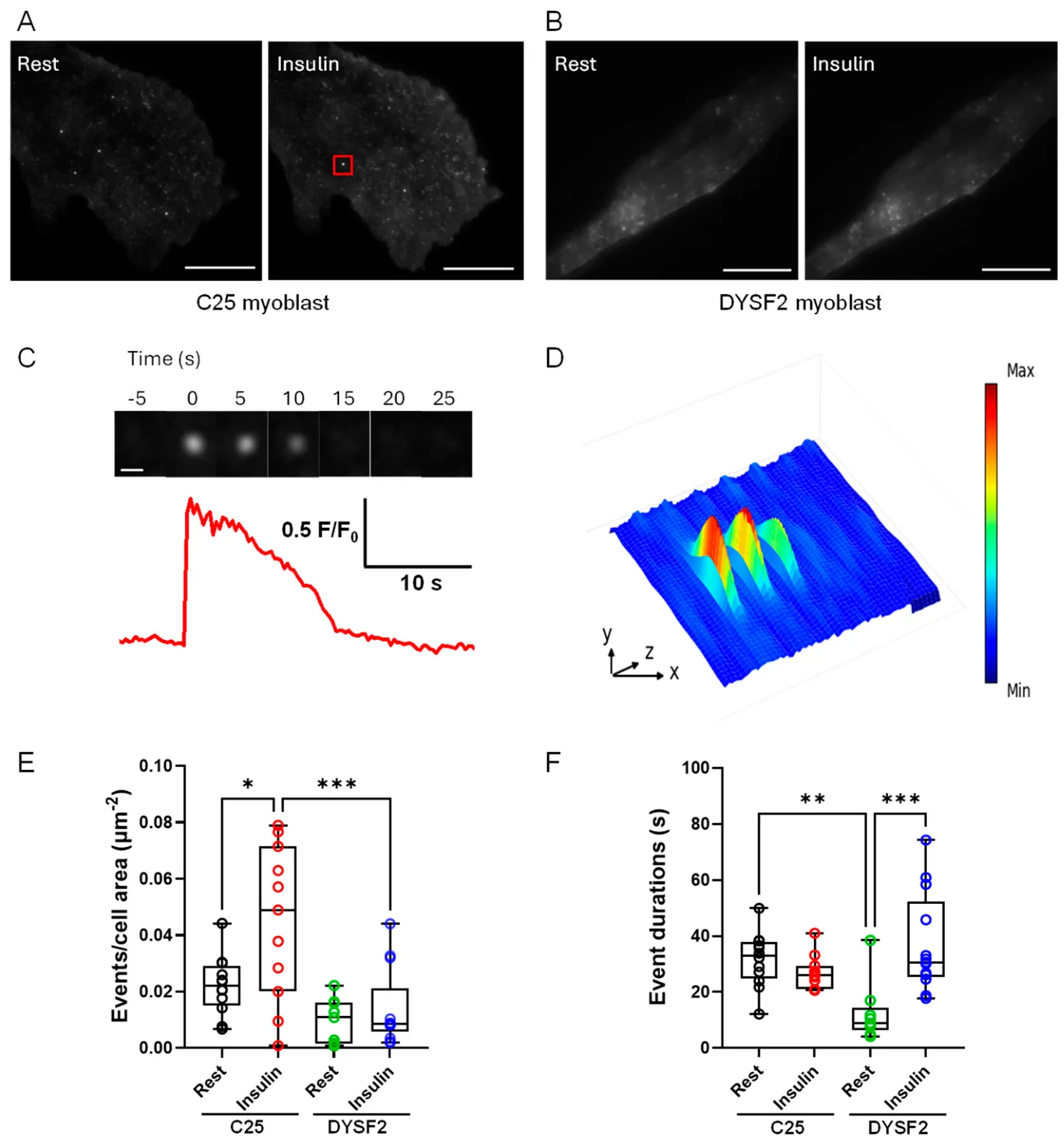
Insulin-induced exocytosis is reduced in DYSF2 myoblasts. Both C25 and DYSF2 myoblasts were transfected with IRAP-pHluorin. Exocytosis was monitored 48 h post-transfection under resting conditions (Rest) or following stimulation with 100 nM insulin using TIRFM. (**A**,**B**) A TIRFM image of a C25 (**A**) and DYSF myoblast (**B**) in resting conditions (**left** panels) stimulated with 100 nM insulin (**right** panels). Scale bar = 5 μm. The red square in the right panel of (**A**) indicates a selected exocytosis event. (**C**) **Upper**: Sequence of frames depicting the exocytosis event indicated by the red square in (**A**) (**right** panel). Scale bar = 0.25 μm. **Bottom**: Fluorescence intensity profile of the exocytosis event. (**D**) Heat map showing the fluorescence profile of the event displayed in (**C**). The x-, y-, and z-axes represent time, spatial position along the analyzed region, and fluorescence intensity, respectively. (**E**) Comparison of the number of exocytosis events between C25 and DYSF2 myoblasts under resting conditions or after 100 nM insulin stimulation. (**F**) Durations of individual exocytosis events. Data points represent values from individual cells across at least three independent cultures. Horizontal lines indicate means with SEM. Statistical significance indicated as * *p* < 0.05, ** *p* < 0.01, *** *p* < 0.001 (ANOVA test followed by Tukey’s multiple comparison test).

### 3.4. Expression of Dysferlin-3HA in DYSF2 Cells Rescues Insulin—But Does Not Fully RescueIonomycin-Induced Exocytosis

To test whether dysferlin expression rescues the exocytotic defect, DYSF2 cells were cotransfected with dysferlin-3HA (expression confirmed in Supplementary Figure S1). As shown in Table 1, dysferlin-3HA increased the number of ionomycin-induced exocytic events in DYSF2 cells by ~5-fold; however, this frequency remained lower than in C25 cells. In contrast, dysferlin-3HA fully restored the number of insulin-induced exocytotic events to levels comparable with wild-type C25 cells. These data suggest that ionomycin and insulin engage distinct vesicle populations whose trafficking defects in DYSF2 cells are differentially rescued by dysferlin.

### 3.5. Insulin-Induced GLUT4 Punctum Trafficking Is Impaired in DYSF2 Myoblasts

To determine whether the impaired IRAP-pHluorin exocytosis observed in DYSF2 myoblasts extends to the trafficking of endogenous GLUT4 to the plasma membrane, we analyzed the density of GLUT4 puncta near the plasma membrane by using immunofluorescence, visualized using TIRFM. Consistent with our previous findings in RCMH myoblasts [25], GLUT4 expression appeared as punctate, vesicle-like structures (Figure 4A), likely representing both exocytosed GLUT4 and GSVs tethered near the plasma membrane. Stimulation with 100 nM insulin for 30 min significantly increased surface-proximal GLUT4 puncta in control C25 cells, whereas this response was absent in DYSF2 myoblasts (Figure 4B).

In a subset of cells, GLUT4 puncta were imaged sequentially using TIRFM and epifluorescence microscopy, and the resulting ratio of punctum density (TIRFM/epifluorescence) was quantified (Figure S2). In control C25 cells, insulin stimulation significantly increased this ratio, consistent with a preferential enrichment of GLUT4 puncta at or near the plasma membrane. In contrast, this insulin-dependent increase was absent in DYSF2 myoblasts.

## 4. Discussion

The present study demonstrates that myoblasts from a patient with dysferlinopathy (DYSF2 cells) exhibit a marked reduction in the frequency of IRAP-pHluorin exocytosis events evoked by both stimuli, compared to control C25 myoblasts. Impaired exocytosis in DYSF2 cells induced by ionomycin occurred despite cytosolic and submembrane Ca^2+^ signals being comparable to those of healthy control C25 cells. However, expression of dysferlin-3HA fully restored insulin-induced exocytosis in DYSF2 cells to levels comparable to C25 controls. In contrast, while dysferlin-3HA expression increased the frequency of ionomycin-induced exocytosis five-fold, this rescue was incomplete and did not reach the levels observed in healthy controls.

The differential rescue observed with dysferlin-3HA suggests that ionomycin and insulin mobilize distinct vesicle pools. Ionomycin, by inducing a broad and sustained increase in cytosolic ${\mathrm{Ca}}^{2+}$, triggers the exocytosis of diverse vesicle populations and organelles, including early endosomes [26] and lysosomes [2]. Consistent with this, the frequency of IRAP-pHluorin exocytosis events in control C25 cells was approximately 8-fold higher following ionomycin stimulation compared to insulin (Table 1).

Exocytotic kinetics are known to vary across distinct organelle populations; for example, the exocytosis of small synaptic-like vesicles in astrocytes occurs 1.5 times faster than that of lysosomes [27]. Similarly, the recruitment of distinct vesicle pools by ionomycin versus insulin may account for their differing exocytotic kinetics. In C25 cells, the exocytotic event duration was 81 ± 7 s following ionomycin stimulation, compared to 27 ± 2 s following insulin stimulation. Distinct kinetic patterns were also observed in DYSF2 cells: ionomycin-induced exocytosis was reduced relative to stimulated C25 controls (Figure 2), whereas insulin-induced exocytosis was elevated relative to unstimulated DYSF2 cells (Figure 3). However, further experiments are required to confirm that insulin and ionomycin indeed mobilize distinct vesicle pools in this context.

Because the exocytosis induced by ionomycin was not fully rescued by dysferlin-3HA, secondary cellular alterations likely influence Ca^2+^-dependent trafficking. Supporting this idea, recent deep-proteomic analyses of muscle tissue from dysferlinopathy patients revealed widespread proteomic remodeling, with over 1000 proteins upregulated and more than 200 downregulated relative to healthy controls, including key regulators of membrane trafficking [7]. Consequently, expressing dysferlin alone may be insufficient to fully restore all disrupted trafficking pathways. In line with this, Chen and Michael [28] recently reported that syntaxin-4 overexpression enhances plasma membrane repair in flexor digitorum brevis fibers from A/J mice (a dysferlinopathy model), suggesting that SNARE-dependent membrane repair can occur independently of dysferlin. However, as the authors noted, the endogenous contribution of dysferlin to membrane repair might be masked or complemented by the adaptive upregulation of other exocytotic proteins. Similar secondary proteomic remodeling could explain why dysferlin-3HA expression failed to fully rescue ionomycin-induced exocytosis in DYSF2 cells.

Two modes of exocytosis have been described for vesicles carrying plasma-membrane proteins: full-collapse fusion and transient fusion (kiss-and-run). In TIRFM imaging of pHluorin-tagged reporters, full fusion appears as discrete events with lateral fluorescence dissipation, whereas kiss-and-run events display rapid fluorescence dimming without lateral diffusion [22,23,24]. In our prior work [18], transient non-lateral events predominated under both basal and stimulated conditions. Because transient fusion typically delivers less cargo to the cell surface than full-collapse fusion [23], it is critical to determine whether impaired IRAP-pHluorin exocytosis leads to a reduction in cell-surface delivery of endogenous GLUT4.

Using TIRFM to image endogenous GLUT4 immunofluorescence, we found that insulin increased GLUT4 punctum density near the plasma membrane in control C25 cells, whereas this response was absent in DYSF2 myoblasts (Figure 4). While this approach cannot distinguish bona fide GLUT4 surface insertion from vesicles tethered beneath the plasma membrane, it demonstrates that insulin-stimulated GSV trafficking to the vicinity of the plasma membrane is impaired in DYSF2 myoblasts.

The impaired insulin-stimulated trafficking of endogenous GLUT4 in DYSF2 myoblasts raises the question of why clinical insulin resistance or overt metabolic dysfunction is not typically reported in dysferlinopathy patients. One possibility is that non-affected or less-damaged muscle groups preserve systemic glucose clearance. In support of this, mouse models demonstrate that GLUT4 levels vary across different muscle types during disease progression. For example, in 10-month-old dysferlin-deficient BLAJ mice, GLUT4 protein content is reduced in the fast extensor digitorum longus muscle yet remains unchanged in the predominantly slow soleus [29]. Similarly, augmented GLUT4 expression and glucose uptake were observed in the quadriceps, but not the tibialis anterior muscle, of 11-month-old dysferlin-deficient B6.129-Dysftm1Kcam/J mice [8]. Proteomic analyses of the quadriceps in these animals further revealed significant reductions in enzymes involved in glycolysis and glycogen metabolism [8], suggesting that chronic dysferlin deficiency induces complex, muscle-specific metabolic remodeling.

Proteomic profiling of affected muscles from patients with dysferlinopathy has likewise demonstrated marked downregulation of proteins involved in glucose and energy metabolism, including enzymes involved in glycogen utilization such as muscle glycogen phosphorylase (PYGM). Reduced PYGM abundance has also been associated with disease progression in dysferlinopathy [7]. Whether these alterations primarily reflect secondary responses to ongoing muscle pathology or represent localized metabolic adaptations remains to be determined.

While the present findings suggest insights into dysferlin-dependent trafficking, certain limitations should be considered. First, although human immortalized myoblasts serve as a valuable cellular model, they do not recapitulate the complex structural and physiological environment of differentiated skeletal myofibers. Second, our study evaluated a single patient-derived cell line (DYSF2). While larger, heterogeneous cohorts are needed to account for phenotypic variability and establish generalizability, our primary objective was mechanistic discovery rather than population-level characterization. By identifying a specific defect in a well-characterized cell line, this study provides preliminary evidence that dysferlin loss may perturb GLUT4 trafficking and exocytotic control. Nevertheless, future studies using differentiated myotubes, additional DYSF variants, and animal models will be essential to define the context-dependent roles of dysferlin in regulated exocytosis.

## 5. Conclusions

In summary, our findings demonstrate that dysferlin plays a differential role in regulating distinct exocytotic pathways in human myoblasts. While dysferlin deficiency impairs both ionomycin- and insulin-induced exocytosis, dysferlin re-expression completely restores the insulin-stimulated pathway while providing only a partial rescue of ionomycin-triggered events. This functional divergence suggests the engagement of distinct vesicle pools with different kinetic profiles and susceptibility to secondary proteomic alterations. Furthermore, the loss of insulin-stimulated GLUT4 translocation to the cell surface vicinity in dysferlin-deficient myoblasts suggests that impaired GLUT4 trafficking might contribute to the complex metabolic remodeling observed in dysferlinopathy muscle. Overall, these results provide an insight into the complex trafficking defects underlying dysferlinopathy and emphasize the need to target both direct dysferlin function and secondary proteomic networks when developing therapeutic strategies.

## Figures and Tables

**Figure 1:**
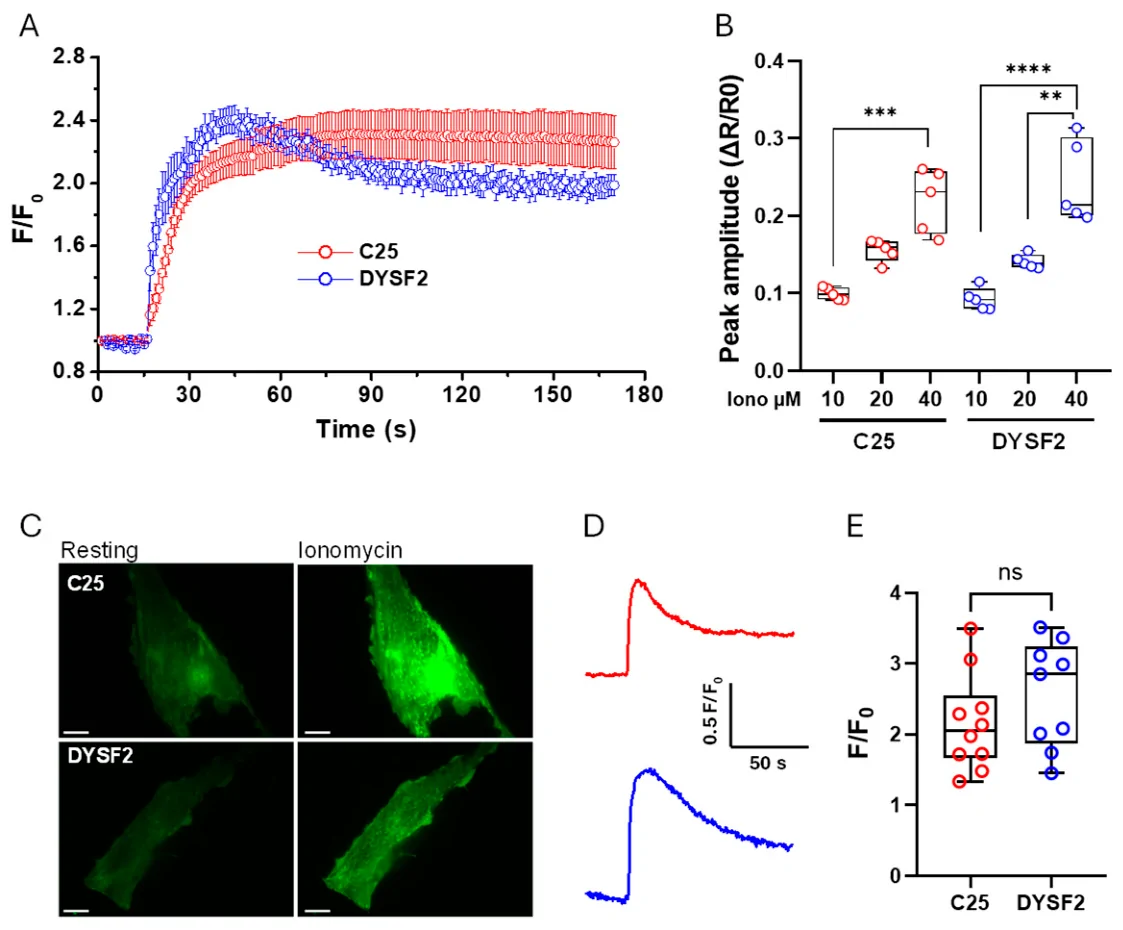
Intracellular Ca^2+^ signals in control C25 and dysferlinopathy DYSF2 myoblasts. (**A**) Time-lapse transients of cytosolic Ca^2+^ signals induced by 10 μM ionomycin in cells loaded with 5 μM Fluo-4; the arrow indicates the time of ionomycin application. Individual dots represent average F/F_0_ value from single cells. Data are expressed as mean ± SEM from three independent cultures (n = 51 cells per cell line). (**B**) Peak Ca^2+^ signal amplitudes (expressed as ΔR/R_0_) in Fura-2-loaded cells following stimulation with 10, 20, or 40 μM ionomycin. Each dot represents the average peak response from a single field of cells. Data are presented as mean ± SEM from five independent experiments. Statistical significance was determined using a one-way ANOVA followed by Tukey’s post hoc test for all pairwise comparisons. * *p* < 0.05, ** *p* < 0.01, *** *p* < 0.001, **** *p* < 0.0001. (**C**) Representative TIRFM images of C25 (**upper** panels) and DYSF2 (**lower** panels) myoblasts expressing Lck-GCAMP3 at rest (**left** panels) and after application of 10 μM ionomycin (**right** panels). (**D**) Examples of ionomycin-induced submembrane Ca^2+^ transients in C25 (red) and DYSF2 (blue) myoblasts. (**E**) Peak Ca^2+^ responses. Each dot represents the maximum F/F_0_ value from a single cell. Data are expressed as mean ± SEM from three independent cultures (n = 10 for C25 and 9 for DYSF2 cells, respectively). No significant differences were found between groups (two-tailed Student’s *t*-test).

**Figure 4:**
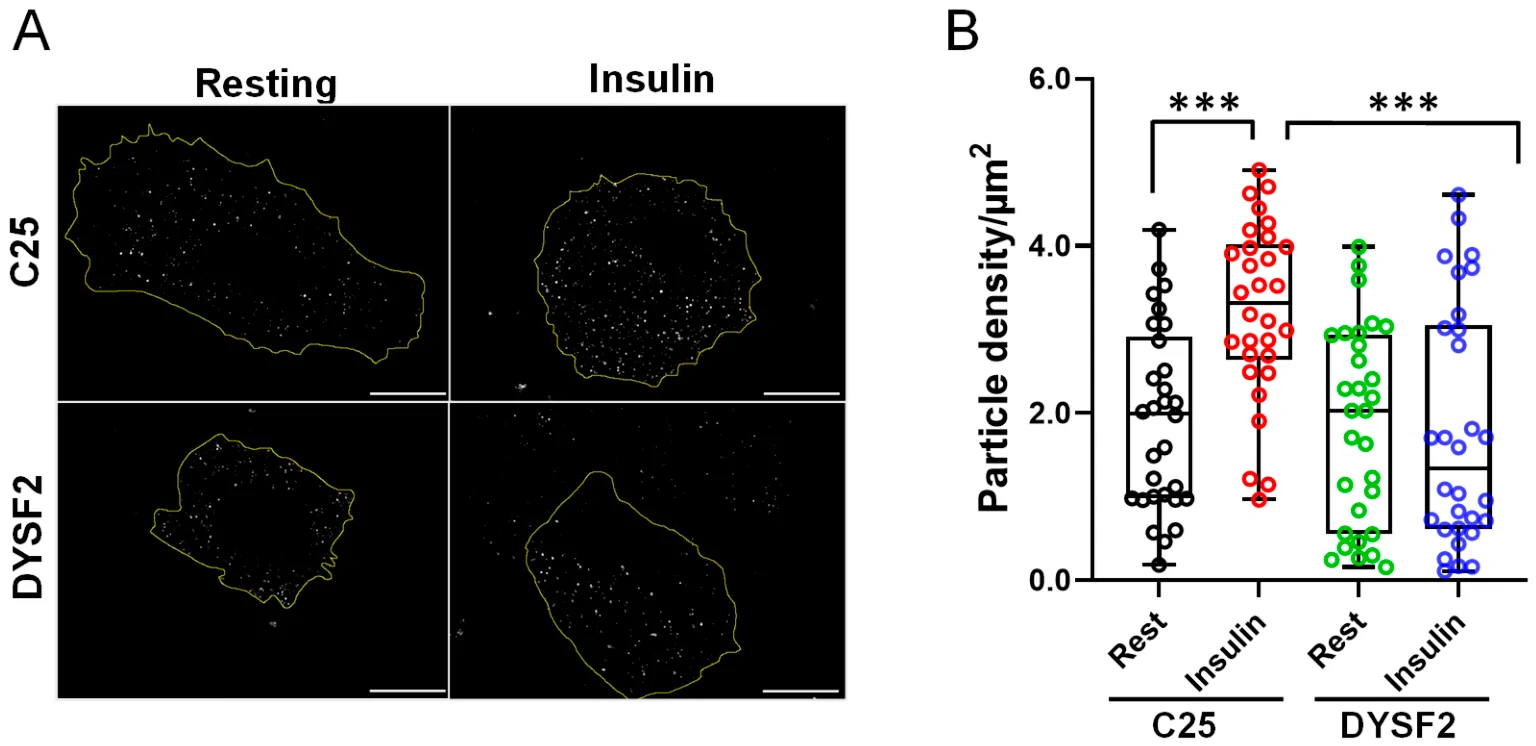
Insulin increases GLUT4 puncta near the plasma membrane in C25 myoblasts but not in DYSF2 cells. Surface-proximal GLUT4 puncta were quantified by immunofluorescence using an anti-GLUT4 antibody in C25 and DYSF2 myoblasts at rest (Rest) or after stimulation with 100 nM insulin for 30 min. Images were acquired by TIRFM, and particle density at the plasma membrane was quantified in Fiji (ImageJ) as described in Methods. (**A**) Representative TIRF images of C25 and DYSF2 cells at rest (**left** panels) or after stimulation (**right** panels) with insulin. Outlines indicate cell perimeters. Scale bar = 5 μm. (**B**) Data points represent individual cells from at least three independent cultures; horizontal lines indicate mean ± SEM. Statistical significance: *** *p* < 0.001 (ANOVA followed by Tukey’s multiple comparisons).

**Table 1:** Expression of dysferlin-3HA in DYSF2 cells restores insulin-induced exocytosis but not ionomycin-induced exocytosis. C25 and DYSF2 cells were transfected with IRAP-pHluorin, and exocytosis was induced with 10 µM ionomycin or 100 nM insulin and monitored by TIRFM. To rescue dysferlin expression, DYSF2 cells were cotransfected with a dysferlin-3HA construct (dysf3HA). Data are means ± SEM. Cell numbers are given in parentheses. Outcome variables did not meet normality (Shapiro–Wilk and D’Agostino–Pearson tests). * *p* < 0.05; *** *p* < 0.0001 compared with C25 cells; ^#^ *p* < 0.05 compared with DYSF2 cells (Kruskal–Wallis followed by Dunn’s multiple-comparisons test).

**IONOMYCIN**					
**Number of Events/Cell Area (µm^−2^)**			**Event Durations (s)**		
C25	DYSF2	DYSF2 + dysf3HA	C25	DYSF2	DYSF2 + dysf3HA
0.35 ± 0.07 (n = 11)	0.01 ± 0.004 *** (n = 11)	0.05 ± 0.02 (n = 6)	81 ± 7 (n = 11)	46 ± 8 * (n = 11)	51 ± 7 (n = 6)
**INSULIN**					
**Number of events/cell area (µm^−2^)**			**Event durations (s)**		
C25	DYSF2	DYSF2 + dysf3HA	C25	DYSF2	DYSF2 + dysf3HA
0.05 ± 0.008 (n = 11)	0.013 ± 0.004 * (n = 10)	0.05 ± 0.01 ^#^ (n = 6)	27 ± 2 (n = 11)	37 ± 5 (n = 10)	39 ± 5 (n = 6)

## Data Availability

The data presented in this study are available on request from the corresponding author.

## Supplementary Materials

The following supporting information can be downloaded at: https://www.mdpi.com/article/10.3390/biomedicines14102207/s1.

## Abbreviations

The following abbreviations are used in this manuscript:

DMEM/M199	Dulbecco’s modified Eagle minimal essential medium/199 medium
GSV	GLUT4-containing vesicle
HBECS	HEPES-buffered extracellular solution
IRAP	Insulin-regulated aminopeptidase
PBS	Phosphate-buffered saline
SNARE	Soluble N-ethylmaleimide-sensitive factor attachment protein receptor
TIRFM	Total internal reflection fluorescence microscopy
