# Supplementary material for: Impaired Regulated Exocytosis and GLUT4 Trafficking in Dysferlin-Deficient Human Myoblasts: A Pilot Study
Source: Biomedicines. 2026 Sep 29;14(10):2207. doi: 10.3390/biomedicines14102207 (PMC13701912; doi:10.3390/biomedicines14102207)
Supplement: Supplementary file 1 [file biomedicines-14-02207-s001.zip › biomedicines-4153923-supplementary.pdf]

Supplementary Figures

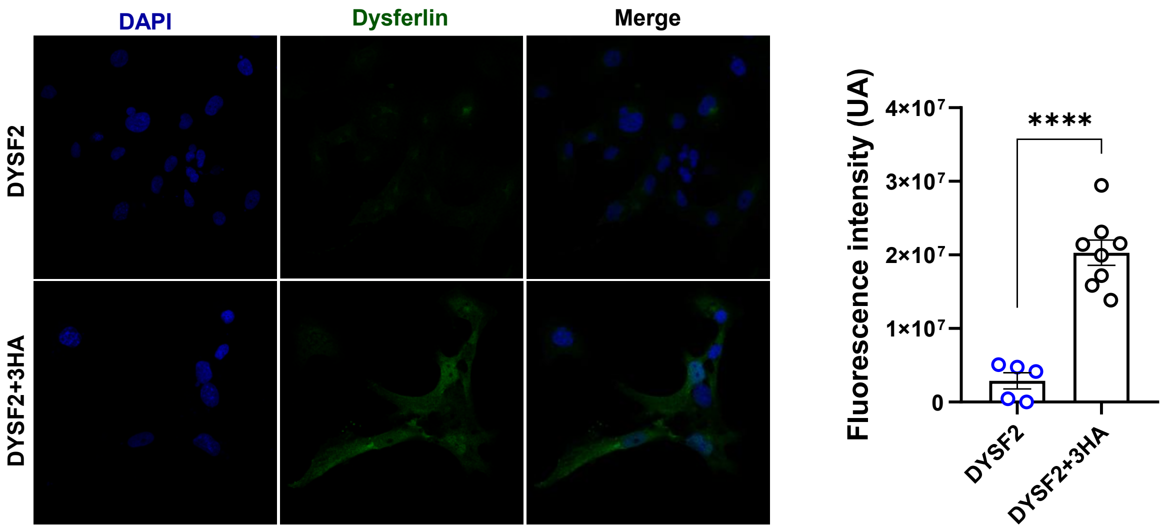

Figure S1. *Dysferlin expression in untransfected and transfected DYSF2 myoblasts*. (A) Representative immunofluorescence images of DYSF2 myoblasts left untransfected or transfected with dysferlin-3HA. Dysferlin was labeled with a rabbit anti-dysferlin primary antibody (Abcam, ab124684; 1:300 dilution) and a Cy2-conjugated anti-rabbit secondary antibody (Jackson ImmunoResearch; 1:1000 dilution). Dysferlin signal is shown in green, and nuclei are counterstained with DAPI (blue). Merged images are shown in the right panels. (B) Quantification of mean dysferlin fluorescence intensity per cell. Data are presented as mean ± SEM. Statistical significance was determined using an unpaired Student's (****P < 0.0001).

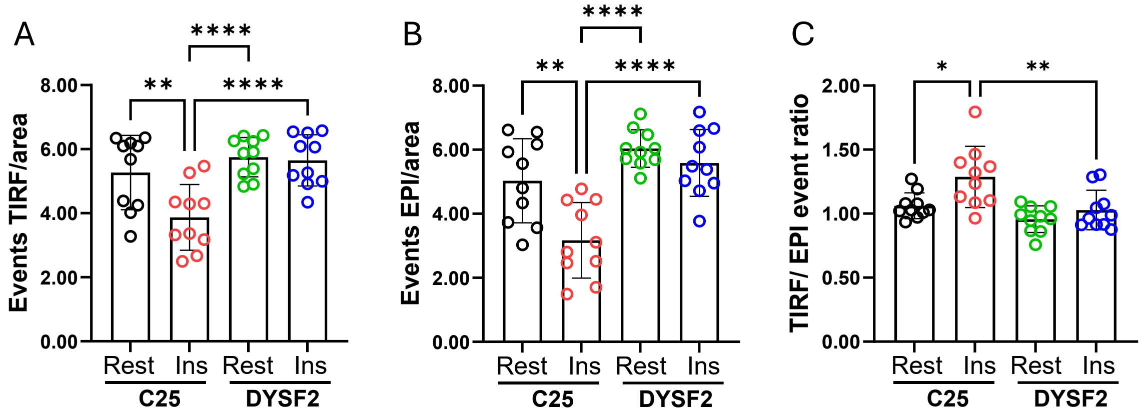

Figure S2. *GLUT4 puncta density measured by TIRFM and epifluorescence microscopy*. GLUT4 puncta were quantified by immunofluorescence using an anti-GLUT4 antibody in C25 and DYSF2 myoblasts under basal conditions (Rest) or after stimulation with 100 nM insulin (Ins) for 30 min. The same cells were imaged sequentially by TIRFM and epifluorescence microscopy (EPI), and puncta densities were quantified in Fiji (ImageJ) as described in the Methods. **(A–B)** GLUT4 puncta density, expressed as the number of detected events normalized to the acquired cell area, measured by TIRFM (A) and EPI (B). **(C)** Ratio of TIRFM to EPI puncta density for each cell, providing a normalized measure of the relative membrane-proximal GLUT4 signal. Each data point represents an individual cell; horizontal lines indicate the mean ± SEM. Statistical significance was assessed using one-way ANOVA followed by Tukey's multiple-comparisons test. $^*p < 0.05$; $^{**}p < 0.01$. Note that in this experimental set, lower absolute puncta densities were detected in C25 cells treated with insulin in both TIRFM and EPI modes compared to baseline; however, the normalized TIRFM/EPI ratio was significantly increased relative to all other conditions.
